# Quantitative Comparison of the Performance of Piezoresistive, Piezoelectric, Acceleration, and Optical Pulse Wave Sensors

**DOI:** 10.3389/fphys.2019.01563

**Published:** 2020-01-14

**Authors:** Hongju Wang, Lu Wang, Nannan Sun, Yang Yao, Liling Hao, Lisheng Xu, Stephen E. Greenwald

**Affiliations:** ^1^College of Medicine and Biomedical Information Engineering, Northeastern University, Shenyang, China; ^2^School of Computer Science and Engineering, Northeastern University, Shenyang, China; ^3^Neusoft Research of Intelligent Healthcare Technology, Co. Ltd., Shenyang, China; ^4^Blizard Institute, Barts and The London School of Medicine and Dentistry, Queen Mary University of London, London, United Kingdom

**Keywords:** pulse wave, sensor, performance, reproducibility, stability, quantitative analysis

## Abstract

The accurate measurement of the arterial pulse wave is beneficial to clinical health assessment and is important for the effective diagnosis of many types of cardiovascular disease. A variety of sensors have been developed for the non-invasive detection of these waves, but the type of sensor has an impact on the measurement results. Therefore, it is necessary to compare and analyze the signals obtained under a range of conditions using various pulse sensors to aid in making an informed choice of the appropriate type. From the available types we have selected four: a piezoresistive strain gauge sensor (PESG) and a piezoelectric Millar tonometer (the former with the ability to measure contact force), a circular film acceleration sensor, and an optical reflection sensor. Pulse wave signals were recorded from the left radial, carotid, femoral, and digital arteries of 60 subjects using these four sensors. Their performance was evaluated by analyzing their susceptibilities to external factors (contact force, measuring site, and ambient light intensity) and by comparing their stability and reproducibility. Under medium contact force, the peak-to-peak amplitude of the signals was higher than that at high and low force levels and the variability of signal waveform was small. The optical sensor was susceptible to ambient light. Analysis of the intra-class correlation coefficients (ICCs) of the pulse wave parameters showed that the tonometer and accelerometer had good stability (ICC > 0.80), and the PESG and optical sensor had moderate stability (0.46 < ICC < 0.86). Intra-observer analysis showed that the tonometer and accelerometer had good reproducibility (ICC > 0.75) and the PESG and optical sensor had moderate reproducibility (0.42 < ICC < 0.91). Inter-observer analysis demonstrated that the accelerometer had good reproducibility (ICC > 0.85) and the three other sensors had moderate reproducibility (0.52 < ICC < 0.96). We conclude that the type of sensor and measurement site affect pulse wave characteristics and the careful selection of appropriate sensor and measurement site are required according to the research and clinical need. Moreover, the influence of external factors such as contact pressure and ambient light should be fully taken into account.

## Introduction

The arterial pulse wave contains much physiological and pathological information and its accurate measurement can improve the diagnosis of cardiovascular disease (Ma et al., 2013; Papaioannou et al., 2016), now a major public health problem worldwide (Yang et al., 2016). Many types of sensors have been developed for the non-invasive detection of pulse waves (Schafer and Vagedes, 2013; Meidert et al., 2014; Stea et al., 2014; Boutry et al., 2015; Kamshilin et al., 2016). One of the earliest devices used for this purpose was the applanation tonometer, first used clinically in 1902 (Mackenzie, 1902; O’Rourke, 2016). Commercial products appeared in 1970, of which the CBM series produced by the Colin Company in Japan were among the most widely used (Kemmotsu et al., 1994). Subsequently, many other types of sensors have been developed and used in a clinical setting. These include piezoresistive and piezoelectric devices as well as photoelectric sensors. Chen et al. (2016) presented an ultra-flexible strain sensor for the long-term measurement of pulse waves. Murphy et al. (2011) proposed a piezoelectric sensor based on polyvinyl difluoride (PVDF) for the measurement of pulse wave velocity (PWV) in hypertensive patients. Clemente et al. (2010) designed a piezo-film-based measurement method to reconstruct the blood pressure waveform. Lovinsky (2006) presented an optical pulse sensor system to monitor arterial oxygen saturation and Loukogeorgakis et al. (2002) proposed a new method for measuring PWV using reflectance photoplethysmography. Li et al. (2018) used photoplethysmography to investigate the changes of arterial waveform characteristics in pregnant women.

In the last few years, some studies have combined different sensor types for pulse acquisition. For example, Huotaril et al. (2013) used electro-mechanical film (EMFi) and photoplethysmographic (PPG) sensors to measure pulse waves from the left forefinger, wrist, and second toe arteries, and compared the pulse wave decomposition parameters between EMFi and PPG to obtain information about arterial elasticity. Wang et al. (2015) combined a pressure sensor with a photoelectric sensor array to make a multichannel device and demonstrated that this device was more effective than previous pulse acquisition platforms. These and other studies demonstrate the increasing use of pulse wave analysis derived from different types of sensors to obtain prognostic and diagnostic information from patients with cardiovascular disease, especially hypertension (Townsend et al., 2015) and are a sign of the increasing need to provide screening of at-risk patients in a primary care setting, thus relieving pressure on specialist centers. By analyzing pulse wave characteristics, these devices can be used to obtain useful diagnostic information such as PWV, peripheral resistance, vascular compliance, and blood flow changes. The above parameters can be used to determine the degree of vascular stiffness, reflect cardiovascular status, and predict the onset or track the progression of cardiovascular disease.

Although all these devices can detect and record the arterial pulse wave, their mode of operation and sensitivity differ, leading to differences in the shape and timing of the pulse wave and thus to variations in their diagnostic effectiveness (Zuo et al., 2016). Therefore, to exploit the advantages of each type of sensor and to optimize their effectiveness, it is necessary to analyze their stability and repeatability, the influence of external factors on their performance as well as their overall design. At present, there is a lack of literature directly comparing the performance of different sensor types.

In this study, we chose the three kinds of sensors that are most widely used in clinical practice, pressure sensor, PPG sensor, and acceleration sensor. In order to verify the influence of contact force the acquired signal, we also added a sensor developed by our laboratory that can measure contact pressure. Firstly, a circular film acceleration sensor which measures the acceleration perpendicular to the skin by detecting the dilation of underlying arteries to record the pulse wave (Elgendi, 2014; Muehlsteff et al., 2015); secondly, a PPG sensor in which a photodiode produces light, some of which is absorbed by blood in superficial vessels below the skin. By detecting the time varying reflection due to blood volume changes during the cardiac cycle, a representation of the pulse wave is obtained (Hertzman, 1937); thirdly, a piezoresistive strain gauge sensor (PESG) designed in our laboratory, which converts pressure signals from the arteries into a change in the strain-dependent resistance, in a bridge circuit to produce a varying voltage which corresponds to the pulse wave signal (Wang et al., 2013; Xu et al., 2014; He et al., 2017); finally, a piezoelectric Millar tonometer which operates in a similar manner. An effective sensor must be robust, stable, and give reproducible signals. Many factors can affect their performance, including the way in which they are applied to the skin, the contact force between the sensor and the skin (Teng and Zhang, 2006), and the measuring site (Lukas et al., 2014; Hartmann et al., 2019). Therefore, we have analyzed the four sensors’ susceptibilities to these external factors and compared their stability and reproducibility.

## Materials and Methods

### Subjects

The study included 60 healthy college students (30 females and 30 males) with mean age 24 ± 2 years, mean height 167.5 ± 5.7 cm, mean weight 59.3 ± 9.2 kg, mean heart rate 69 ± 8 bmp, mean systolic blood pressure 119 ± 9 mmHg, and mean diastolic blood pressure 78 ± 8 mmHg. All participants were fully informed about the study and gave their informed consent. The study was designed in accordance with the Helsinki Declaration and was approved by the local ethics committee. All measurements were taken after 24 h without alcohol or caffeine with an otherwise normal diet. Smokers, and those on any medication were excluded from the study and none of the subjects had exercised vigorously within 1 h before the measurement.

### Protocol

All measurements were performed in a quiet environment after a rest period of 15 min, during which personal information (age, height, weight) was obtained and the study protocol was explained. For the radial artery measurements, subjects were seated with the left arm bent at the elbow to an angle of 90 ± 5° and the forearm resting on a table. Subjects were asked to keep their palms relaxed. Signals were recorded for 30 s from a point above the radial artery near the wrist where the strongest pulse was found (in the order: PESG, tonometer, accelerometer, and optical probe, explained in more detail below), and the study protocol was repeated for each subject three times.

The accelerometer is an inertial device, which records the pulse wave by detecting displacement of the skin over the artery under investigation (Elgendi, 2014). In this study, the accelerometer sensor was interfaced to a multichannel physiological recorder BL-420S (Taimeng Software, Chengdu, China), sampling at a frequency of 1 kHz. The tonometer is a pressure sensor using the principle of applanation tonometry (SphygmoCor, AtCor Medical, Sydney, NSW, Australia) (Crilly et al., 2007a), sampling frequency 128 Hz. The PESG, sampling at 1 kHz was designed and constructed in our laboratory, and held against the skin by a strap. The contact pressure is measured by a resistive element and can be adjusted by tightening or loosening the strap by means of a screw. The optical sensor contains an infrared-emitting diode and a phototransistor to detect the light and acts as a photoplethysmograph (Jingfan Technology, Tianjin, China) (von Wowern et al., 2015). The sampling frequency was 70 Hz.

The tonometer was used as a hand-held device whereas the other sensors were held in contact with the skin by means of a strap. Due to the limitations of the sensor measurement principles, not all sites could be measured by all sensors. For example, the PESG can only measure the pulse wave of the carotid and the radial arteries owing to the length of the strap and sensor size limitations, the finger area was too small for the sensor, and the femoral artery, even when traversing the inguinal ligament, was too deep to give good quality signals. The sites measured by each sensor are listed in Table 1.

**TABLE 1:** Measurement sites and the corresponding pulse sensors.

	**Site**			
**Sensor**	**Radial**	**Carotid**	**Femoral**	**Digital**
PESG	√	√	—	—
Tonometer	√	√	√	—
Optical probe	√	√	—	√
Accelerometer	√	√	√	√
				

The experimental design was as follows. First, as shown in Table 2, at the wrist, three levels of contact force (light, medium, and heavy) were applied during acquisition of the subject’s pulse wave from the left radial artery. The contact force was measured by the PESG, optical probe, and accelerometer. In this study, light contact was defined as a force between 0.6 and 1 N, medium in the range (1.6–2 N), and heavy, in the range (2.8–3.2 N). Due to the shape of the tonometer it was not feasible to measure contact force. Therefore, when using the tonometer, we were only able to subjectively judge the contact force. Then, to allow comparison of the four sensors, each was used in turn to obtain pulse waves (the type of sensor, acquisition position, pressure size, and measurement times are shown in Table 3). Finally, the pulse wave was acquired from the left radial artery by the optical sensor at two different ambient light levels, i.e., full room lighting (LED lighting), external daylight only.

**TABLE 2:** The sequence of pulse wave acquisition at the three levels of contact force (where, L, M, and H represent light, medium, and heavy force, respectively).

**Sensor**	**PESG**			**Tonometer**			**Optical probe**			**Accelerometer**		
Site	Radial			Radial			Radial			Radial		
Force	L	M	H	L	M	H	L	M	H	L	M	H
Number of	1			1			1			1		
measurements												

**TABLE 3:** The sequence of pulse wave acquisitions (^∗^ marks the measurement position for the reproducibility and stability analysis).

**Sensor**	**PESG**	**Tonometer**	**Optical probe**	**Accelerometer**
Site	Radial^∗^, carotid	Radial^∗^, carotid, femoral	Radial^∗^, carotid, digital	Radial^∗^, carotid, digital, femoral
Force	Medium	Medium	Medium	Medium
Number of measurements	3	3	3	3

Reproducibility refers to the variability between results measured under the same conditions from a given subject during repeated measurements. Two observers measured pulse waves sequentially using the same probe types for the analysis of inter-observer reproducibility. To assess intra-observer reproducibility, the pulse wave was measured three times, in the radial artery only, by the same observer, with no interval between the measurements (Chen et al., 1997; Crilly et al., 2007b). Stability is the ability of a sensor to maintain its performance parameters for a period of time. For the stability assessment, the pulse wave was measured three times, again only in the radial artery, by the same observer in 1 h. For each subject the pulse wave for each sensor on the radial artery was measured by the same observer for 30 s, and the sequences of pulse waves were ensemble averaged to one cycle. Then, the time and amplitude of the pulse wave were normalized to the range 0–1. The dynamic time warping (DTW) algorithm, which is sensitive to the amplitude of the pulse signal, was used to analyze the similarity of the two normalized waveforms (Jeong and Jayaraman, 2015).

**FIGURE 1:**
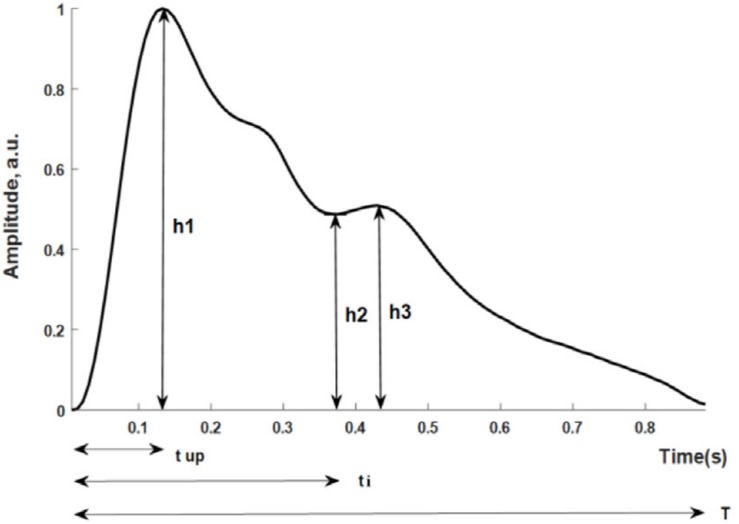
Parameters directly determined from the measured pulse wave.

Figure 1 shows the pulse wave parameters included in this study. They are defined as follows: (*h*_1_-normalized amplitude of the systolic maximum, *h*_2_-normalized incisura minimum); (*h*_3_-maximum following the dicrotic notch); *t*_up_-time interval from foot to systolic maximum; *t*_*i*_-time interval from foot to incisura; and *T*-cardiac cycle. Five additional parameters were derived from the measurements: *h*_2_/*h*_1_, *h*_3_/*h*_1_, *t*_up_/*T*; *K*-value; SER-spectral energy ratio.

The *K*-value is a characteristic quantity based on the amplitude of the pulse wave, and is defined as (Luo et al., 2006):

(1) $$\,K=\frac{P_{\text{m}}-P_{\text{d}}}{P_{\text{s}}-P_{\text{d}}}$$

where *P*_m_ is mean arterial pressure of *P*(*t*), defined over a cardiac cycle (*T*) as:

(2) $$P_{\text{m}}=\frac{1}{T}\,\sum P\,\left(t\right)\,\Delta \,\left(t\right)$$

and *P*(*t*) is the arterial pressure at point *t* in the cardiac cycle; *P*_d_ is diastolic blood pressure; and *P*_s_ is the systolic blood pressure.

Spectral energy ratio has been used to describe differences between pulse wave shapes (Thakker and Vyas, 2010). The instantaneous frequency spectrum is defined as:

(3) $$S_{k}\,\left(k\right)=F\,\left[W\,\left(n\right)\cdot x\,\left(n\right)\right]$$

(4) $$W\,\left(n\right)=\frac{1}{2}\cdot \left\{1-\cos\left[2\,\pi \,n/\left(N-1\right)\right]\right\}$$

where *F*[] represents the Discrete Fourier transform, *W*(*n*) is a Hanning window function, and *x*(*n*) is the pulse wave signal.

The power spectrum is defined as:

(5) $$S_{x\,x}\,\left(k\right)=\bar{S_{x}^{\prime }\,\left(k\right)\cdot S_{x}\,\left(k\right)}=\bar{{\left|S_{x}\right|}^{2}}$$

where$\,S_{x}^{\prime }\,\left(k\right)$ is the complex conjugate of *S*_*k*_(*k*).

The spectral energy within the range of 0 to *i* Hz is defined as:

(6) $$E\,\left(i\right)=\int_{0}^{i}S_{x\,x}\,\left(f\right)\,\text{d}\,f.$$

In this analysis, we introduce the SER:

(7) $$\text{SER}=\frac{E_{1}}{E_{2}}=\frac{\int_{0}^{5}S_{x\,x}\,\left(f\right)\,\text{d}\,f}{\int_{0}^{20}S_{x\,x}\,\left(f\right)\,\text{d}\,f}$$

where *E*_1_ is the energy within the frequency range 0 and 5 Hz; *E*_2_ is the total energy between 0 and 20 Hz.

### Statistical Analysis

Data analysis was performed in MATLAB (MathWorks, Natick, MA, United States), first, the pulse wave signals measured by the four sensors were pre-processed to remove baseline drift and reduce noise. The baseline drift was removed by applying “db7” wavelet decomposition (Xu et al., 2005), and de-noised by decomposing the pulse signal at level 4 and eliminating all the details (Mallat, 1989). Then, feature points (onsets, peak points, and dicrotic notch points of the pulse wave) were extracted by windowing methods to further analyze the pulse wave parameters (Yao et al., 2017).

All statistical analysis was performed using SPSS (version 19.0). All subsequently tested variables were assessed for the normality of their distribution using the Shapiro–Wilkes test. Values were expressed as mean ± standard deviation. Pearson’s correlation analysis and determination of coefficient of variation were performed to assess the effect of external factors on the pulse wave. Analysis of variance (ANOVA) was performed to examine whether different sensors would affect the calculation of dynamic time warp distance. The intra-class correlation coefficient (ICC) has been used as a standard for measuring the reproducibility of continuous data in several clinical studies (Papaioannou et al., 2007). ICC was calculated for the assessment of intra-observer reproducibility and stability (Bland and Altman, 1986). Values of *p* < 0.05 were considered to indicate statistical significance. It is generally considered that an ICC value > 0.75 implies good reproducibility; 0.4 < ICC ≤ 0.75, medium reproducibility; and ICC ≤ 0.4, poor reproducibility (Perloff et al., 1993).

## Results

### Factors Influencing Sensor Performance

#### The Effect of Contact Force on the Pulse Wave

**FIGURE 2:**
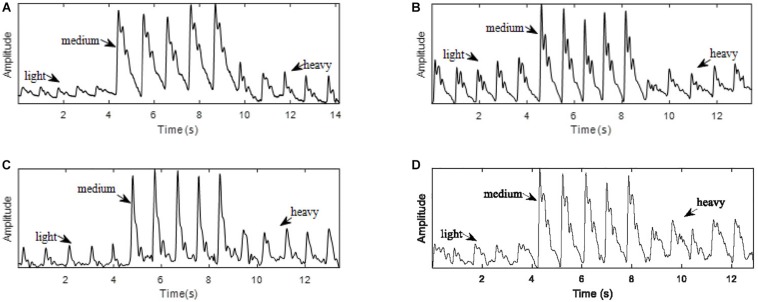
Pulse waves acquired with light, medium, and heavy contact forces. **(A)** PESG. **(B)** tonometer, **(C)** optical sensor, and **(D)** accelerometer.

**FIGURE 3:**
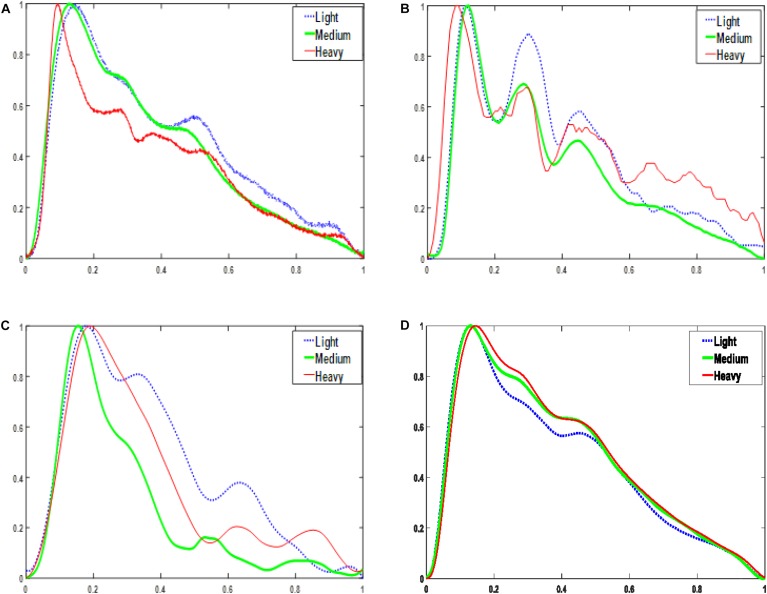
Normalized one-period pulse waves acquired at three magnitudes of contact force. **(A)** PESG. **(B)** tonometer, **(C)** optical sensor, and **(D)** accelerometer.

As shown in Figure 2, for each sensor type, when the contact force was increased from light to medium, the peak-to-peak amplitude of the signal increased. However, a further increase in the application pressure reduced the amplitude. In general, medium contact force was found to produce the highest signal amplitude and, compared with those obtained under lighter and heavier contact forces, the waveform difference of the four sensors under medium pressure is small. Given the dependence of signal amplitude on contact force, it is important to carefully control this to optimize the signal to noise ratio. Figure 3 shows the effect of changing the probe contact pressure on the normalized waveforms from each sensor type. As shown in Figure 3, the optical probe signals are the most strongly affected by probe contact pressure.

#### The Effect of Measurement Site on the Pulse Wave

As shown in Table 4, the coefficients of variation of the parameters (*t*_up_/*T*, *h*_2_/*h*_1_, and *K*-values) for the radial pulse wave were less than those of the carotid pulse wave, which revealed that the quality of the measurements from the radial artery was better, at least in terms of more consistent beat-to-beat stability. In general, the coefficients of variation for carotid artery measurements with all probes were higher than those of the radial artery. With the probes from which measurements were obtained, the coefficients of variation for the digital and femoral arteries were less than the corresponding carotid and radial values. The accelerometer was able to obtain pulse wave signals from all four measurement sites, and the tonometer worked satisfactorily at the radial and femoral sites. The analysis below was confined to data from the radial artery because this location is more convenient for measurements of contact force.

**TABLE 4:** Coefficients of variation for the derived pulse wave parameters, acquired by the four kinds of pulse sensors from the four measuring sites (missing values relate to probe/site combinations for which measurements were impracticable).

	**Sensor**				
				**Optical**	
**Site**	**Parameter**	**PESG**	**Tonometer**	**probe**	**Accelerometer**
Radial	*t*_up_/*T*	0.039	0.014	0.028	0.016
	*h*_2_/*h*_1_	0.048	0.044	0.047	0.059
	*K*	0.044	0.027	0.055	0.039
Carotid	*t*_up_/*T*	0.058	0.019	0.036	0.022
	*h*_2_/*h*_1_	0.067	0.045	0.078	0.105
	*K*	0.101	0.031	0.057	0.041
Digital	*t*_up_/*T*	–	–	0.019	0.013
	*h*_2_/*h*_1_	–	–	0.034	0.066
	*K*	–	–	0.025	0.032
Femoral	*t*_up_/*T*	–	0.008	–	0.017
	*h*_2_/*h*_1_	–	0.074	–	0.065
	*K*	–	0.038	–	0.033

#### The Effect of Ambient Light Intensity on the Pulse Wave

For the optical sensor, it was found that the amplitude of the main peak was significantly correlated with the intensity of the ambient light (*r* = 0.26, *P* < 0.05). Since the other sensors detected either pressure or acceleration, the ambient light intensity had no effect.

### Stability Analysis

As shown in Table 5, the ICC results obtained by the tonometer were in the range 0.88–0.98. For the accelerometer the corresponding figures were 0.82–0.91, so the stability of these two sensors was good. The ICC results obtained for the PESG were between 0.64 and 0.86, suggesting a moderate level of stability and the results obtained for the optical sensor ranged between 0.46 and 0.80, implying only poor stability.

**TABLE 5:** ICC of the derived parameters.

	**Parameter**					
**Sensor type**	***t*_up_**	***t*_*i*_**	***t*_up_/*T***	***h*_2_/*h*_1_**	***h*_3_/*h*_1_**	**SER**
PESG	**0.86**	**0.79**	0.72	0.68	0.65	0.64
Tonometer	**0.88**	**0.91**	**0.91**	**0.92**	**0.88**	**0.98**
Optical probe	**0.80**	0.72	0.70	0.64	0.46	0.51
Accelerometer	**0.88**	**0.82**	**0.87**	**0.89**	**0.91**	**0.84**

*Bold text indicates the items with better results and performance.*

### Reproducibility Analysis

#### Intra-Observer Reproducibility

Table 6 shows that the ICC of the frequency and time domain parameters obtained from the tonometer and accelerometer was >0.75, implying good reproducibility. The ICC results obtained from the optical probe and PESG were as follows: ICC (SER) was <0.75, ICC (*t*_up_, *t*_*i*_, and *t*_up_/*T*) was >0.75, and ICC (*h*_2_/*h*_1_, *h*_3_/*h*_1_) was between 0.40 and 0.75, indicating poor reproducibility. The shapes of the acquired waves were assessed by measuring the magnitude and timing of several fiducial points and further compared by a DTW approach (von Wowern et al., 2015). The intra-observer reproducibility was assessed by the DTW method as described in below.

**TABLE 6:** ICC of the parameters in the time and frequency domains for intra-observer reproducibility.

	**Parameter**					
**Sensor type**	***t*_up_**	***t*_*i*_**	***t*_up_/*T***	***h*_2_/*h*_1_**	***h*_3_/*h*_1_**	**SER**
PESG	**0.82**	**0.88**	**0.91**	0.62	0.64	0.71
Tonometer	**0.80**	**0.93**	**0.89**	**0.92**	**0.89**	**0.78**
Optical probe	**0.80**	**0.82**	**0.80**	0.42	0.50	0.61
Accelerometer	**0.85**	**0.88**	**0.92**	**0.95**	**0.96**	**0.84**

*Bold text indicates the items with better results and performance, having good reproducibility.*

#### Inter-Observer Reproducibility

Table 7 shows that the ICC of the frequency and time domain parameters obtained from the accelerometer was >0.80, implying good reproducibility. The ICC results obtained from the tonometer sensor, optical probe, and PESG were as follows: ICC (*t*_up_/*T*, *h*_2_/*h*_1_, *h*_3_/*h*_1_, and SER) of PESG was <0.75, indicating moderate reproducibility. ICC (*t*_up_, *t*_*i*_, *t*_up_/*T*, and SER) of tonometer was >0.80, ICC (*t*_up_, *t*_*i*_, *t*_up_/*T*, *h*_2_/*h*_1_, and SER) of optical probe and tonometer was >0.80, indicating good reproducibility.

**TABLE 7:** ICC of the parameters in the time and frequency domains for inter-observer reproducibility.

	**Parameter**					
**Sensor type**	***t*_up_**	***t*_*i*_**	***t*_up_/*T***	***h*_2_/*h*_1_**	***h*_3_/*h*_1_**	**SER**
PESG	**0.9**	**0.87**	0.43	0.61	0.47	0.55
Tonometer	**0.96**	**0.83**	**0.80**	0.52	0.65	**0.86**
Optical probe	**0.92**	**0.89**	**0.92**	**0.81**	0.66	**0.96**
Accelerometer	**0.92**	**0.90**	**0.80**	**0.80**	**0.82**	**0.97**

*Bold text indicates the items with better results and performance, having good reproducibility.*

#### Morphological Analysis of the Entire Waveform

In this study the DTW method was used to compare two pulse trains captured successively by a single observer, from which intra-observer reproducibility was quantified.

The DTW distance is defined as (Izakian et al., 2015):

$$\mathrm{DTW}\left(X_{n},Y_{m}\right)=d\left(x_{n},y_{m}\right)+\min\{\mathrm{DTW}\left(X_{n-1},Y_{m}\right),$$

(8) $$\mathrm{DTW}\left(X_{n-1},Y_{m-1}\right),\mathrm{DTW}\left(X_{n},Y_{m-1}\right)\}$$

where *d* is a distance matrix in which each element *d*(*x*_*i*_, *y*_*j*_) represents the distance between two sample points (*x*_*i*_, *y*_*j*__)_ from the signal *X*_*n*_ = [*x*_1_, *x*_2_,…, *x*_*n*_] and *Y*_*m*_ = [*y*_1_, *y*_2_,…, *y*_*m*_].

**FIGURE 4:**
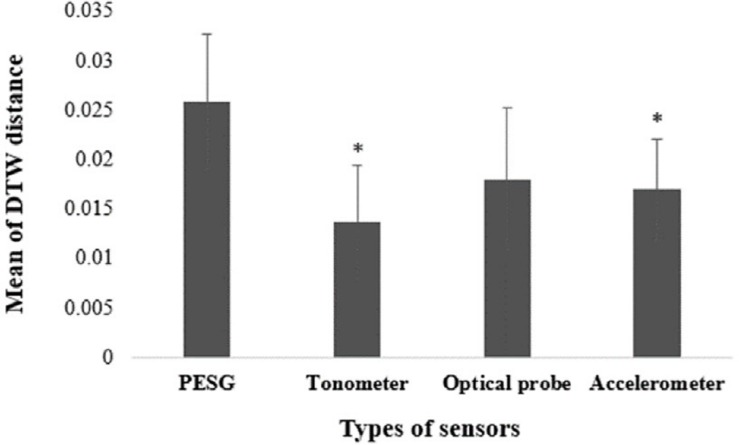
Mean of DTW distance measured with the four sensors [^∗^ marks significant differences in comparison with PESG (*p* < 0.05)].

To test if there were significant differences in the calculated mean and standard deviation of the DTW distance using ANOVA, 20 groups of left radial artery data from the same subject were collected by the same observer using the four sensors, as shown in Figure 4. The mean value of the DTW distance of the PESG was the largest, at 0.026 ± 0.007; the optical sensor ranked second, at 0.018 ± 0.007; the accelerometer ranked third, at 0.017 ± 0.005; and the tonometer was the smallest, at 0.014 ± 0.006. The ANOVA results showed that the optical sensor was not significantly different when compared to the PESG on DTW distance (*p* = 0.08), and that the DTW values for the tonometer and accelerometer were significantly lower than that of the PESG (*p* < 0.05). This suggests that the intra-observer reproducibility of the overall waveform shape acquired by the tonometer was the highest, the accelerometer ranked second, the optical sensor ranked third, and the PESG device had the lowest reproducibility.

#### Analysis of the Timing and Amplitude Parameters

**FIGURE 5:**
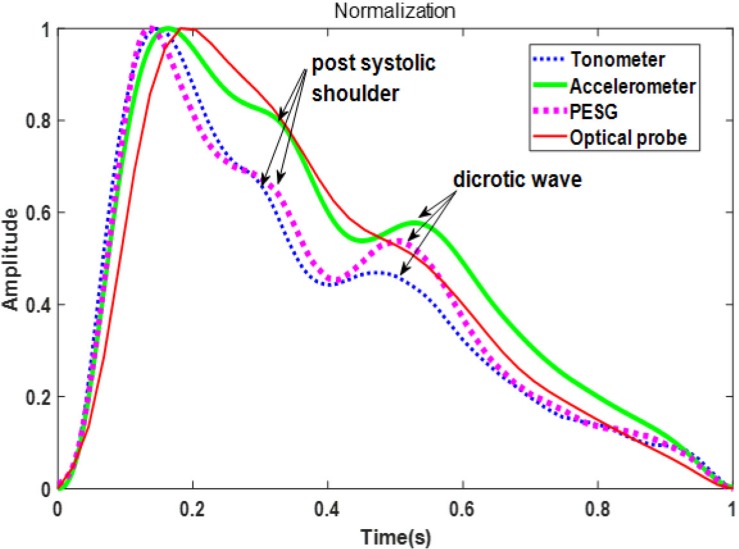
Comparison of the normalized waveforms from each sensor.

Figure 5 shows the waveforms from the four sensors recorded from one beat of the same subject (the sensors were used in the sequence: PESG, tonometer, optical sensor, and accelerometer) and normalized in amplitude and time. It can be seen that the dicrotic notch in the waves acquired by the PESG, tonometer, and accelerometer was more prominent than that acquired by the optical sensor. The post-systolic pressure wave acquired by the optical probe was much less prominent that that generated by the other probes. The detailed differences between the waveforms acquired by the four sensors are discussed below.

**FIGURE 6:**
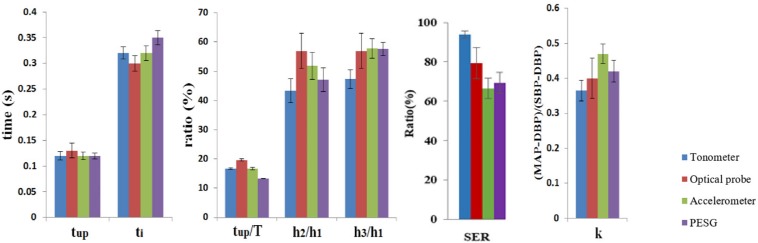
Comparison of the mean ± SD of the parameters obtained by all four sensors for all subjects.

As shown in Table 8, the mean and low SD values of the parameters obtained from the radial artery by four sensors for 60 subjects, and by the relatively small error bars (Figure 6), the beat-to-beat variability of *t*_up_, *t*_up_/*T* was small, the coefficients of variation being <3%, and the reproducibility of these parameters was good. A slightly larger variability was shown by *t*_*i*_, SER, and *k*-values. The coefficients of variation were 6–8%, and the reproducibility was medium. A larger variability was observed for *h*_2_/*h*_1_. The coefficients of variation were 10–15%, and the reproducibility was poor. The similarity of the feature parameters acquired by the four sensors was poor, and the measurement difference of *h*_2_/*h*_1_, *h*_3_/*h*_1_, SER, and *k*-values was obvious.

**TABLE 8:** Comparison of the mean ± SD of the parameters obtained by all four sensors for all subjects.

	**Parameter**						
**Sensor type**	***t*_up_**	***t*_*i*_**	***t*_up_/*T* (%)**	***h*_2_/*h*_1_ (%)**	***h*_3_/*h*_1_ (%)**	**SER (%)**	***k***
PESG	0.12 ± 0.01	0.35 ± 0.01	13.21 ± 0.1	47.14 ± 4.02	57.58 ± 2.3	69.26 ± 5.26	0.42 ± 0.03
Tonometer	0.12 ± 0.01	0.32 ± 0.01	16.68 ± 0.3	43.30 ± 4.1	47.33 ± 3.2	93.93 ± 1.87	0.37 ± 0.03
Optical probe	0.13 ± 0.02	0.30 ± 0.01	19.63 ± 0.34	56.98 ± 5.97	56.98 ± 5.97	79.28 ± 7.9	0.40 ± 0.06
Accelerometer	0.12 ± 0.01	0.32 ± 0.01	16.69 ± 0.49	51.76 ± 4.64	57.79 ± 3.28	66.51 ± 5.23	0.47 ± 0.03

## Discussion

We analyzed signals recorded over the radial, carotid, digital, and femoral arteries of 60 subjects with four sensors, and extracted seven pulse wave parameters: *t*_up_, *t*_*i*_, *t*_up_/*T*, *h*_2_/*h*_1_, *h*_3_/*h*_1_, *K*-values, and SER. The reason for selecting these parameters is that *t*_up_, *t*_*i*_, and *t*_up_/*T* can reflect the degree of atherosclerosis (Weber et al., 2010); *h*_2_/*h*_1_ and *h*_3_/*h*_1_ can reflect the level of peripheral resistance (Korpas et al., 2009); the *K*-value can reflect changes in blood flow variables, such as peripheral resistance, vascular wall elasticity, and blood viscosity, and thus can independently detect the presence of cardiovascular disease. Finally, SER has been used to discriminate between healthy subjects and those with gastrointestinal disorders, which are characterized by significant differences in the SER power spectra (Thakker and Vyas, 2010).

### External Factors Influencing Sensor Performance

We also analyzed the effect of some external factors (i.e., contact force, measuring site, and ambient light intensity) that affect signal shape and quality. The PESG can measure the contact force quantitatively, while the contact force exerted by the other sensors can only be assessed subjectively by the operator, unless mounted above a pressure sensor, as described in this study. We observed significant differences in pulse wave morphology among the three levels of applied force (light, medium, and heavy). As the force increases from light to medium, peak to peak amplitude of the signal increases. However, a further increase of contact force reduces the peak to peak amplitude. These findings are consistent with those of Thakker et al. (2010) and Teng and Zhang (2004). Further theoretical work by Teng and colleagues has shown that not only is the amplitude of the PPG signal dependent on probe pressure but also is its timing with respect to the arterial pulse. They found that pulse transit times increased with increasing pressure, until the transmural pressure was zero and that there was little further change as the probe pressure exceeded the arterial pressure and the transmural pressure thus became negative (Teng and Zhang, 2006, 2007). Given the strong effect of probe contact pressure on the shape and timing of the arterial pulse wave it is clearly important to establish an optimal and widely accepted standard for measurements of this type. This has been suggested by others for PPG measurements (Grabovskis et al., 2013), but there have been few reports describing the effect of contact pressure on tonometric recordings. Indeed, signal optimization in commercial tonometry systems is left in the hands of the experienced operator.

Not surprisingly, we observed that the quality of pulse wave measurement is affected by the measuring site. The finding is consistent with those of Hartmann et al. (2019). The stability of the pulse wave obtained from the carotid artery is worse than that of the radial artery (Adji et al., 2006). The accelerometer can obtain pulse wave signals at multiple measurement sites, and the tonometer is able to obtain pulse waves from the radial and femoral arteries. However, for PWV dual-channel acquisition devices the usefulness of radial artery measurements is limited, because carotid–radial PWV is not a good marker of large artery stiffness or, therefore, of general vascular health. Furthermore, because of the limitations of the experimental equipment in this study, the analysis approach adopted here, while of value to those concerned with analyzing the shape of the pulse wave, is of limited utility for PWV measurements. Nevertheless, there is value in using the radial pulse in PWV measurements because it has been shown that when combined with signals from the ankle, this ankle brachial “PWV” (abPWV) is significantly correlated with more direct measurement of large artery elasticity, such as carotid femoral PWV (Sugawara et al., 2005), and may even be more strongly correlated with overall cardiovascular health than carotid–femoral PWV. Therefore, because abPWV is easier to measure with simple and inexpensive equipment, it could be more suitable than carotid–femoral PWV for large-scale screening of at-risk populations in spite of the obvious theoretical drawback that abPWV is not a true velocity: in the sense that the distance used to calculate the velocity from the pulse transit time is not the actual distance traveled by the pulse.

Another important use of the radial artery pulse, especially when measured by tonometry, is as an adjunct to peripherally obtained blood pressure in monitoring hypertensive patients. Using the so-called generalized inverse transfer function, this approach can compensate for the amplification of the peripheral pressure pulse and not only obtain aortic pressure but also visualize the central pulse waveform (O’Rourke et al., 2001). It is also used in the measurement of PWV clinically (Yoon et al., 2000; O’Rourke and Seward, 2006; Salvi et al., 2008).

It was found that the optical sensor is affected by ambient light intensity, but available PPG systems commonly compensate electronically for changes in ambient light by alternately sampling the ambient light and the biological signal and subtracting the former from the latter (Rajaguru and Prabhakar, 2015) so this problem is minimized, although not entirely absent. Therefore, in practice it is prudent, when using optical probes, to minimize the problem by carrying out measurements under constant artificial light.

Most of the energy in cardiovascular signals (excluding ECG) is found at frequencies below 20 Hz (Wang and Xiang, 2002; Wei and Chow, 2007). Therefore, a sampling frequency of no <40 Hz should be adequate to faithfully reproduce all information of pathophysiological interest. Thus, for the optical sensor, the manufacturer’s choice of 70 Hz, although unusual, is more than adequate to reproduce the signal faithfully enough for analyzing its shape. Of course, this sampling rate is not adequate when measuring pulse transit times in the order of a few tens of milliseconds. In this study we have not measured pulse transit times although in future work concerning PWV the sample rate would have to be increased or another device used.

### Stability and Reproducibility

In terms of stability and reproducibility, the tonometer is superior to the other sensors, but it still has some shortcomings. For instance, unlike wristband or finger sensors, it normally functions as a hand-held device, thus requiring the operator to keep the probe static during the measurement period. As mentioned above, the effect of pressure to the skin and underlying artery has a strong effect on the signal characteristics. The acceleration sensor is integrated with a multi-channel data acquisition system (Nelson et al., 2010) and can simultaneously obtain pulse wave signals from multiple measurement sites, while the hardware supplied with the other sensors used in this study allows single channel use only.

Morphological analysis shows that there is a significant difference between the waveforms acquired by the four sensors. This is not unexpected as they are measuring different physical properties. The optical sensor, for instance, is detecting blood volume changes in micro-vessels (Challoner, 1979; Wang et al., 2014), although PPG pulse sensors produce their strongest signals when positioned over large superficial arteries, which suggests that under these conditions their movement greatly augments the micro-vessel signals (Loukogeorgakis et al., 2002).

Photoplethysmographic technology has widespread clinical application and has been used in commercially available medical devices for measuring oxygen saturation, vascular assessment, assessing autonomic function, and also detecting peripheral vascular disease (Allen, 2007; Gil et al., 2010). The accelerometer detects skin movement where the skin is minimally loaded, and the two pressure sensors derive their signals directly from the blood pressure in the large arteries on which they are pressing. Piezoresistive and piezoelectric sensors can be easily implemented in various wearable devices, which can detect subtle physiological signal changes before and after exercise (Luo et al., 2016).

The PESG is used in the quantitative analysis of pulse characteristics and can investigate changes in the shape of the pulse waves under varying contact pressure (Luo et al., 2012; Bae et al., 2013; Chu et al., 2014), while the contact force exerted by the other sensors can only be assessed subjectively by the operator. The acceleration sensor has been applied to simultaneously obtain pulse wave signals at multiple measurement sites (Gardosi et al., 1991), and wearable optical pulse sensors have been commercially developed (Tamura et al., 2014). To fully exploit the advantages of each type of measurement system it would be useful to develop a versatile data acquisition unit compatible with a variety of sensor types including but possibly not limited to those described here.

## Conclusion

The above results show that firstly, the effect of contact pressure, measuring site, and ambient light on the pulse wave should be considered when carrying out measurements on patients. Secondly, comparison of the four kinds of pulse wave sensors shows that, overall, the performance of the tonometer is the best, the accelerometer ranks second, the PESG, third, and the optical sensor is the poorest. Finally, there is significant difference among the four sensors in their waveform shapes and the timing and amplitude parameters.

In terms of stability and reproducibility, the tonometer is superior to the others although it normally functions as a hand-held device. Unlike wristband or digital sensors for instance, it requires the operator to keep the probe static during the measurement period. Furthermore, the tonometer is not equipped to measure the contact pressure so this can only be assessed subjectively by the operator. However, in practice, different sensor types could be used, perhaps in combination according to the measurement site and the nature of the required signal analysis and in this way, the advantages of each can be more easily exploited.

### Recommendation

Researchers can reasonably select sensor types according to their own experimental requirements. Obviously, the main factor determining choice of sensor type and measurement position is what type of cardiovascular pathology is being investigated: central or peripheral disease, response to treatment acute measurements, or longer-term monitoring. In addition, when the data are being recorded, external factors that affect the experimental results, such as contact pressure, measurement position, ambient light, etc., need to be considered to rationally design the experimental environment. For the assessment of arterial stiffness, by measuring PWV, consensus documents have been published which specify the optimal measuring sites, how to measure path length, and acceptable levels of repeatability (Laurent et al., 2006; Wilkinson, 2010; Van Bortel et al., 2012). However, in other areas, such as the general field of analyzing the shape of the pulse wave or the assessment of peripheral arterial disease there remains a lack of authoritative guidelines.

## Data Availability Statement

All datasets generated for this study are included in the article/supplementary material.

## Ethics Statement

The studies involving human participants were reviewed and approved by the Ethics Committee of Medicine and Biomedical Information Engineering College, Northeastern University, Shenyang, China. The patients/participants provided their written informed consent to participate in this study.

## Author Contributions

HW and NS contributed to the conception and design of the study and wrote the manuscript. LX and SG contributed to the manuscript revision, read, and approved the submitted version of the manuscript. YY, LW, and LH provided some suggestions. All authors contributed to the manuscript revision, read, and approved the submitted version of the manuscript.

## Conflict of Interest

LX was partly employed by Neusoft Research of Intelligent Healthcare Technology, Co. Ltd., Shenyang, China. The remaining authors declare that the research was conducted in the absence of any commercial or financial relationships that could be construed as a potential conflict of interest.
